# Social Capital, Political Efficacy and Chinese Residents’ Willingness to Participate in Food Safety Governance

**DOI:** 10.3390/ijerph192013147

**Published:** 2022-10-13

**Authors:** Nianyu Du, Chuanmei Zhang, Jin Qin, Liangqiang Jiang, Zongshuo Yin, Mo Chen

**Affiliations:** 1 College of Economics, Qufu Normal University, Rizhao 276827, China; 2 Research Center for Food Safety and Agriculture Green Development, Qufu Normal University, Rizhao 276827, China

**Keywords:** food safety governance, social capital, political efficacy, residents’ willingness to participate

## Abstract

Residents play an important role as one of the main actors in food safety governance. To build a pattern of food safety risk co-governance, the positive and effective participation of residents is vital. This study first establishes a comprehensive analysis framework combining social capital theory and political efficacy theory. Data from a survey of 714 residents in Shandong Province, China, were analysed through structural equation modelling and fuzzy-set qualitative comparative analysis (fsQCA) to examine the causal relationship between residents’ willingness to participate and its driving factors. The results indicated that: (1) reciprocity norm, institutional trust and social engagement have significant positive effects on willingness to participate; (2) political efficacy has a partial mediating effect in the relationship between social capital and willingness to participate; (3) fsQCA findings have four solutions to achieving residents’ strong willingness to participate; reciprocity norm, institutional trust and political efficacy are the core elements that affected residents’ high willingness to participate, whereas social engagement and sociodemographic variables are the non-core variables. Therefore, we put forward suggestions for improving residents’ willingness to participate in food safety governance, including improving the appeal expression and feedback mechanism, cultivating residents’ social capital and paying attention to the superposition effect of social capital and political efficacy.

## 1. Introduction

Governments worldwide commonly manage food safety, and their purpose is to deal with the information asymmetry in the food field and the market failure caused by public goods [1]. However, government failures widely occur, and the field of food safety governance is no exception. In this context, food safety governance must rely on the active participation of all social forces, especially consumer regulation [2]. Food safety is related to the most immediate interests of every resident. Allowing residents to participate in food safety governance is in line with the principle of people’s sovereignty, ensuring that the people directly exercise their democratic power. Through such participation, the supervision of food production enterprises can be strengthened, and the level of food safety assurance can be improved. Therefore, residents’ participation in food safety governance has become an important measure for many countries to solve government failures. The Chinese government has introduced different policies and laws that encourage residents to participate in food safety governance by means of economic incentives and policy guidance. In 2011, the Food Safety Commission of the State Council of the People’s Republic of China has brought forth the Guidelines for Establishing the Report-and-Reward System of Food Safety, suggesting that a reward system for food safety should be in place. In 2015, the amended Food Safety Law of the People’s Republic of China established the Report-and-Reward System of Food Safety in legal form, underscoring that all organizations and individuals have the right to report illegal acts against food safety. In 2016, Measures for the Administration of Food and Drug Complaints and Reports encouraged the public to report food safety issues. However, in developing countries like China, due to the relative lack of food safety knowledge and the relatively low enthusiasm for political participation, residents’ participation in food safety governance is low. Surveys have shown that on average, only 22% of consumers participate in food safety governance through complaints, claims, or whistleblowing when faced with food safety issues [3]. How to promote residents’ participation in food safety governance effectively is the bottleneck in food safety co-governance currently faced by Chinese governments.

Empirical studies show that many factors affect residents’ willingness to participate in food safety governance [4,5,6,7,8]. For example, Yin et al. reported that Chinese residents’ participation attitude, subjective norms, perceived behaviour control and ethics had significant positive impacts on their intention to report food safety incidents [4]. Zhu et al. found that self-efficacy and subjective norms affect consumers’ intention to communicate food safety risks with stakeholders [5]. Yi and Lin showed that online social capital has a significant positive impact on residents’ willingness to exchange food safety information on social media. People with more online social capital are more interested in searching for and sharing food safety-related information [6]. Meng et al. developed a questionnaire on consumer psychological capital with the four dimensions of self-efficacy, resilience, hope and optimism to measure the degree of consumer participation in food safety social co-governance [7]. Chen et al. found that residents with high education, high income and good health have higher psychological capital for participating in food safety governance [8]. From the perspective of economics, food safety has the characteristics of public goods and externality. From the perspective of the collective action dilemma, Su et al. explored the reasons for the failure of the Chinese government’s policy to reduce relevant costs to promote consumer participation in food safety governance. The results show that consumers’ participation in food safety governance is low due to the existence of the collective action dilemma caused by the free rider phenomenon [3]. Studies have shown that social capital centred on social trust, social engagement and social networks is the key to solving the collective action dilemma [9,10]. However, to the best of our knowledge, the literature that uses social capital to explain residents’ willingness to participate in food safety governance is rare, and there is no literature with Chinese residents as the research object.

As one of the social actors of food safety governance, residents’ participation in food safety governance is a right granted by the government, which is essentially the orderly political participation of residents. Political efficacy is about the subjective perception that personal political behaviour affects government or politics [11]. It affects the willingness, intensity and behaviour choices of citizens’ political participation and has a significant predictive effect on citizens’ political participation [12]. How political efficacy affects residents’ participation in food safety governance is an academic issue worthy of in-depth study, and we developed a theoretical model that integrates social capital and political efficacy. On the basis of the survey data of 714 qualified samples in China in 2022, the driving factors of residents’ willingness to participate were tested with the help of structural equation modelling. In addition, fuzzy-set qualitative comparative analysis (fsQCA) was used to analyse the configuration to clarify the triggering antecedents of residents’ high willingness to participate.

Compared with the existing literature, the contribution of this study is twofold. Firstly, we studied the impacts of residents’ social capital and political efficacy on their willingness to participate, expanded the application of social capital in the field of food safety governance and provided a new perspective for understanding the internal driving factors of residents’ participation in food safety governance. Secondly, we considered that multiple paths may trigger residents’ high willingness to participate, and traditional quantitative research cannot explain the complex causal relationships between multiple antecedents [13]. Hence, we introduced fsQCA to explore the configurations that trigger residents’ high willingness to participate.

The rest of this study is arranged as follows: The second part is a literature review on the relationship between social capital, political efficacy and residents’ willingness to participate. The third part presents the questionnaire design, measurement methods and descriptive statistics. The fourth part introduces the reliability and validity of the questionnaire, model fitting results and research hypothesis testing. The fifth part uses fsQCA to test the configurations of residents’ willingness to participate. The last part discusses the main conclusions, policy implications drawn by and limitations of this study.

## 2. Research Hypotheses and Analytical Framework

### 2.1. Social Capital

Bourdieu first proposed the concept of social capital [14]. Putnam creatively applied it to public governance and pointed out that the characteristics of social organisations such as trust, norms and networks can improve the efficiency of society by promoting cooperation [15]. After more than 30 years of research and development, the connotation of social capital has become increasingly rich. Social norms, social networks and social trust are recognised as the core elements [16,17,18]. Previous studies confirmed that social capital reduced opportunistic behaviour by promoting communication and cooperation, and formed norms of mutual trust [19]. Therefore, reciprocity, fairness and behavioural norms affect people’s public participation and determine the quality of public participation and the efficiency of solving public problems [16]. Food safety has the attribute of public goods, and a free ride phenomenon exists in the process of residents’ participation in governance. There is an urgent need for relevant theories to solve this collective action problem. On the basis of social capital theory proposed by Putnam, this study believes that reciprocity norm, social engagement and trust are important factors affecting residents’ willingness to participate. The specific analysis follows.

#### 2.1.1. Reciprocity Norm

Social norms are social rules that groups can operate, including formal constraints such as laws and regulations and informal constraints such as accepted codes of conduct, obligations, cooperation and reciprocity [20]. As a general relationship based on morality, the norm of reciprocity has the effect of restraining and regulating members’ interaction behaviour. When both parties in a transaction clearly understand the principle of equal exchange of responsibilities and obligations and reach a social consensus of ‘a favor for you today is made in the tacit understanding that it will be returned someday’ [21], people can expect others to make the same contribution. In the research on personal knowledge-sharing motivation in the virtual community, social interaction, trust and reciprocity norms have an impact on individual knowledge-sharing behaviour [19]. Diedrich et al. came to a similar conclusion: trust and reciprocity can significantly increase the perceived benefits of residents and ensure long-term positive outcomes for marine protected areas [22]. In general, the mutual trust relationship among residents promotes awareness of responsibilities, norms and sanctions and can effectively restrain free-rider behaviour in collective actions. Accordingly, this study proposes the following hypothesis:

**H1.**  *Reciprocity norm has a significant positive effect on residents’ willingness to participate in food safety governance*.

#### 2.1.2. Social Engagement

Putnam believes that citizen participation in networks can help promote social cooperation. Through extensive community participation, people form reciprocity relationships in continuous exchanges and infinite games [15]. People in organisations and groups often form relationships with those around them because of work, beliefs and interests [23]. These relationships involve trust, which leads to cooperation [24]. In social groups such as volunteer organisations, social engagement plays an important role in promoting and stimulating the public’s awareness of participation [16]. It has strong explanatory power for citizen participation behaviours such as election participation and interest expression [25]. High-quality social capital will increase the opportunities for consumers to understand organic food through participating in community activities; both relational and cognitive capital have positive impacts on the knowledge-sharing behaviour of community members and strengthens community members’ perception of the value of organic food [26]. In this study, social engagement includes participation in informal groups such as community health lectures, community volunteer activities, fellow villagers’ associations and interest associations. Accordingly, this study proposes the following hypothesis:

**H2.**  *Social engagement has a significant positive effect on residents’ willingness to participate in food safety governance.*

#### 2.1.3. Trust

Trust is an important part of social capital [15]: it is the moral and value foundation that maintains the sustainable and stable cooperation of members of social organisations [27]. Trust determines the degree to which an individual trusts others to behave as expected [28]. In an organisation with a large number of people, collective action fails when people do not believe that others will participate in the creation of public goods [28]. However, when people have a high level of mutual trust and believe that others will participate in cooperation, then opportunities for the creation of social public goods will increase [29]. That is, the higher the level of trust in an organisation, the greater the likelihood of cooperation. Luhmann (1979) divided trust into interpersonal trust and institutional trust [30]. The former is the trust established by people in their interactions with one another, and the latter is the trust in the government. Interpersonal trust helps reduce barriers to information transfer, reduces transaction costs [28] and increases spontaneous social engagement behaviour [31,32]. Institutional trust mainly refers to the confidence of actors in the material and environmental stability of society [33]. The difference from individual institutional trust is related to the individual’s understanding of institutional commitment and the judgment of the possibility of realising commitment [34]. He et al. confirmed through research that both interpersonal trust and institutional trust positively affect farmers’ willingness to participate in environmental governance [35]. Accordingly, this study proposes the following hypotheses:

**H3.**  *Institutional trust has a significant positive effect on residents’ willingness to participate in food safety governance.*

**H4.**  *Interpersonal trust has a significant positive effect on residents’ willingness to participate in food safety governance.*

### 2.2. Political Efficacy

Political efficacy is an individual’s feeling about one’s political ability, and it is a psychological reflection that taking political action can affect politics or government [11]. Political efficacy is the determinant of political behaviour [36]. According to expectation theory, an individual judges that the greater the possibility of achieving a certain result or purpose through personal efforts, the stronger the incentive force, and the greater the possibility of putting it into action in reality [37]. Numerous studies have confirmed that personal political efficacy plays a positive role in political participation [38,39]. Residents’ participation in food safety governance is a political behaviour, and political efficacy is one of the important factors in predicting residents’ willingness to participate. Accordingly, this study proposes the following hypothesis:

**H5.**  *Political efficacy has a significant positive effect on residents’ willingness to participate in food safety governance.*

### 2.3. Social Capital, Political Efficacy and Residents’ Willingness to Participate in Food Safety Governance

The experience of successfully participating in community group activities may lead individuals to believe that they can exert influence over a political environment [23]. Members of voluntary associations and community groups have the opportunity to deal with political issues. Moreover, the empirical evidence in such political activities leads them to believe that they can have an effective political impact [23]. Broad social participation helps develop personal skills, such as communication and organisational skills, which are important factors in influencing an individual’s political participation [40]. Individuals in groups or environments with a high sense of community will trust the government to listen to their concerns [23]. Therefore, residents with rich social capital believe that they can influence the government and will receive a positive response from the government.

Research has confirmed that organisational members with a high level of trust will have higher participation expectations and are more likely to feel the influence of public policies; that is, they will have a higher sense of political efficacy [27]. Norm-formed reciprocity expectations have enhanced this process; social engagement networks can reduce the cost of participation and enhance the ability of and interest in political participation [41]. Accordingly, the following assumptions are made:

**H6.**  *Reciprocity norm has a significant positive effect on residents’ willingness to participate in food safety governance through the intermediary mechanism of political efficacy.*

**H7.**  *Social engagement has a significant positive effect on residents’ willingness to participate in food safety governance through the mediating mechanism of political efficacy.*

**H8.**  *Institutional trust has a significant positive effect on residents’ willingness to participate in food safety governance through the mediating mechanism of political efficacy.*

**H9.**  *Interpersonal trust has a significant positive effect on residents’ willingness to participate in food safety governance through the mediating mechanism of political efficacy.*

**Figure 1:**
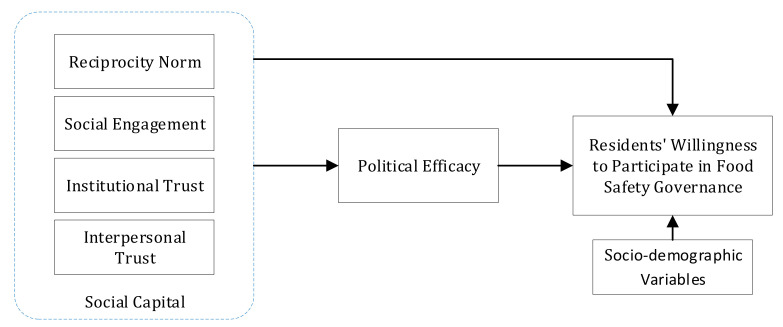
Model diagram of residents’ willingness to participate in food safety governance.

In summary, reciprocity norm, social engagement, institutional trust, interpersonal trust and political efficacy should be strong predictors of residents’ willingness to participate in food safety governance. Among them, political efficacy plays a mediating role between social capital and residents’ willingness to participate. In addition, residents’ social capital, political efficacy and willingness to participate in governance are individual-level variables that will be affected by variables such as individual characteristics and experience. On the basis of existing literature and research, this study introduces sociodemographic variables including gender, age, income and education as the control variables [42]. From this background, a theoretical model that shows the factors that affect residents’ willingness to participate in food safety governance can be established (Figure 1).

## 3. Research Design

### 3.1. Measurement of Variables

To ensure that the measurement of the variables was reasonable and effective, a study questionnaire was designed on the basis of mature scales and previous research results. The formal scale involved 6 constructs and 21 related observable variables, including willingness to participate in food safety governance, reciprocity norm, social engagement, institutional trust, interpersonal trust and political efficacy. All variables were rated on 5-point Likert scales (1 = completely disagree/unwilling; 2 = relatively disagree/unwilling; 3 = usually; 4 = relatively agree/willing; 5 = agree/willing). All measurement items were adapted from existing literature [43,44,45,46,47,48,49]. These items were adjusted, demonstrated and tested in combination with China’s national conditions. Appendix A shows the measurement items and references for each construct.

### 3.2. Research Method

In this study, SEM and fsQCA were used to investigate the driving factors of residents’ willingness to participate in food safety governance and the relationships between them.

#### 3.2.1. Structural Equation Modelling

Social capital involves multiple dimensions, and political efficacy and residents’ willingness to participate are subjective feelings that are difficult to measure. Thus, we used SEM for analysis. SEM includes two parts: the structural model and the measurement model. The structural model is used to reflect the relationships between constructs, and the measurement model is used to reflect the relationships between constructs and measured variables [50]. The specific equation form is as follows:

(1) $$\begin{array}{c} \eta =b\eta +\gamma \xi +\zeta \end{array}$$

(2) $$\begin{array}{c} Y=\Lambda_{y}\eta +\varepsilon \end{array}$$

(3) $$\begin{array}{c} X=\Lambda_{x}\xi +\sigma \end{array}$$

Equation (1) is the structural model, and Equations (2) and (3) are the measurement models. η and ξ denote endogenous constructs and exogenous constructs, respectively; b and γ are the relational matrices of the corresponding variables; Y and X denote observed variables of endogenous constructs and exogenous constructs, respectively; $\Lambda_{x}$ and $\Lambda_{y}$ are factor-loading matrixes for observed variables; and ζ, ε and σ are the error terms.

#### 3.2.2. Fuzzy-Set Qualitative Comparative Analysis

FsQCA is a method based on set relations that realises causal inference through a fuzzy data set. It is an alternative to the traditional quantitative analysis method [51]. This method assumes that many possible solutions or approaches will lead to the same result [13], that is, residents’ willingness to participate in food safety governance can be explained by different combinations of multiple factors. In addition, some factors may be necessary or sufficient conditions for residents’ willingness to participate [13,52].

FsQCA includes data calibration, simplified solutions and the interpretation of results [13]. In data calibration, the original data are converted into fuzzy sets, and the dependent variables (residents’ willingness to participate) are reset as qualitative results [53]. The calculation results are divided into complex, reduced and intermediate solutions. The complex solution includes all possible combinations. The reduced solution includes important conditions that cannot be omitted in any configuration. The intermediate solution is a part of the complex solution including the reduced solution [13]. The intermediate solution reports the core and non-core elements of the solution at the same time. The elements with a strong causal relationship with residents’ willingness to participate are the core elements, and the elements with a weak causal relationship are the non-core elements [54]. The ‘don’t care’ result does not work in the configuration [13].

### 3.3. Data Sources and Descriptive Statistics

The data were collected from 16 cities in Shandong Province, which is located in the eastern coastal area of China, from January to March 2022. The eastern, central and western regions of Shandong Province are characterised by major differences in development that reflect the imbalanced regional development in China [55]. The survey used in the 16 cities in Shandong Province can describe the social capital stock of Chinese residents and their willingness to participate in food safety governance. The specific research method was as follows. Two to three sample sites were randomly selected from each city. The specific research sites were supermarkets and farmers’ markets in urban areas of the target cities. The third resident who came into sight was selected as the interview object to improve the randomness of the sample. The survey questionnaire was completed by conducting face-to-face interviews between trained investigators and respondents. Adults who were over 18 years old, the main household food purchaser and willing to participate in the survey were the respondent criteria. Since the willingness of Chinese residents to participate in food safety governance is generally low at present [2], we only surveyed food purchasers who might be more concerned about food safety. Their willingness to participate in food safety governance is of greater value for improving food safety. Firstly, in January 2022, 70 urban residents were selected in Rizhao City, Shandong Province, for a preliminary survey. The survey plan and questionnaire items were adjusted and improved on the basis of the preliminary survey. In February–March 2022, a formal survey was carried out in the target cities using the improved survey plan. Finally, a total of 800 questionnaires were collected in this survey. After excluding invalid questionnaires such as those with missing data, 714 valid questionnaires were obtained, with a recovery rate of 89.25%.

The basic information of the surveyed are presented in Table 1. Among the study respondents, 390 were female (54.6%) and 324 were male (45.4%), showing that in China most food buyers are female [8]. With regard to the age groups, the 20- to 29-year-olds accounted for the most (26.5%), followed by 40- to 49-year-olds (20.6%) and 30- to 39-year-olds (19.8%). In terms of household income, the largest group was USD 14,830–44,490/year (39.8%), followed by USD 7415–14,830/year (36.0%). Additionally, most respondents (57.6%) had a college or undergraduate degree, while those with a high school or secondary school degree accounted for 22.3%.

## 4. SEM Analysis

### 4.1. Reliability and Validity Analysis

In this study, SPSS25.0 was used for data processing, and AMOS22.0 was used for confirmatory factor analysis and model estimation. The standardised loading measures the internal consistency of each construct, and a value greater than or equal to 0.5 is acceptable. The composite reliability (CR) of constructs is used to judge the internal quality of the model. Generally, CR above 0.7 is considered ideal intrinsic quality [56], and studies considered CR greater than 0.6 to be acceptable [57]. Table 2 shows the results. The standard loadings of all observed variables exceeded a statistically significant level (*p* < 0.05). Except for interpersonal trust, 0.680, which is slightly lower than 0.7, the CRs of other constructs are greater than 0.7, indicating that the intrinsic quality of the scale was ideal.

The design of this survey questionnaire was based on the existing theories and previous research results (see Appendix A), and it was finally determined after several rounds of expert demonstrations. Therefore, it has good content validity. The average variance extracted (AVE) is used as a measure of convergent validity. Generally, the AVE must be greater than 0.5 [56], although in some studies, the threshold is set to 0.4 [57,58].

As shown in Table 2, except for the AVE of interpersonal trust, which is 0.432, slightly lower than 0.5, the AVEs of the constructs were greater than the threshold of 0.5. Thus, the scale met the requirements of convergent validity. discriminant validity is measured by comparing the square root of the AVE of all constructs and the Pearson correlation coefficient of that construct with other constructs. The results are shown in Table 3. The square root of the AVE of all constructs on the main diagonal was larger than the rest of the values, indicating ideal discriminant validity. In summary, the scale of this study had high reliability and validity and could be used for further analysis.

### 4.2. Common Method Variance

Common method variance is a problem when using the questionnaire method to collect data. Although this survey was filled out anonymously, it did not conform to the procedures for controlling common method variance. To test the seriousness of this problem, we carried out Harman’s single-factor test. All items of the questionnaire were subjected to unrotated factor analysis, and the variance explained by the first principal component was 22.95%, which is less than the recommended value of 50% [59,60]. Thus, common method variance was not a serious problem in this study.

### 4.3. Test of Proposed Research Model

This study used a total of 11 indicators in 3 aspects, absolute fitness index, relative fitness index and information index, to test the fit of the structural equation model. The covariation relationships between the error terms were added. The final fitting results are shown in Table 4. Except for the general RMR index, the fitting values of the other indicators met the ideal requirements. The overall fit of the structural equation model was good, and the next step of hypothesis testing could be performed.

### 4.4. Hypothesis Testing

**Figure 2:**
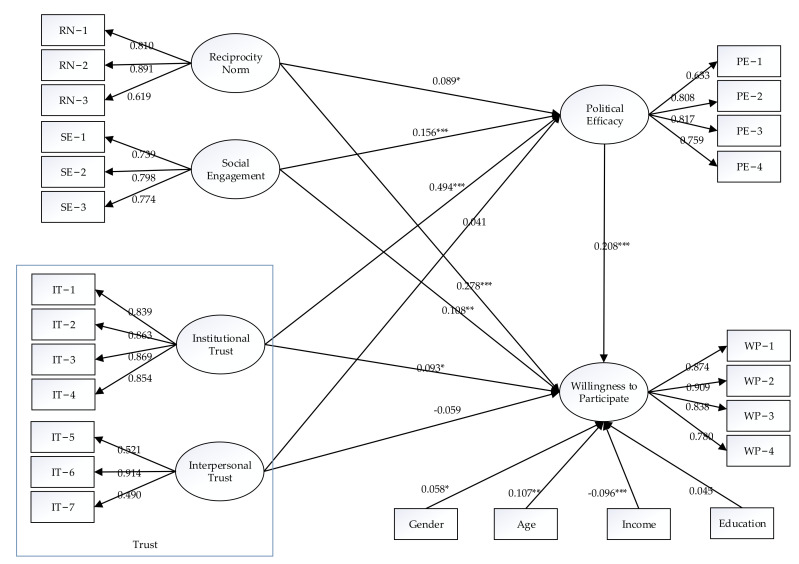
Path results. Structural equation model with standardised path coefficient and significance level; *, ** and *** indicate significance at the 10%, 5% and 1% significance levels, respectively; covariance is allowed between all independent variables.

The path results (see Figure 2) and hypothesis test results (see Table 5) were obtained using AMOS software. After controlling for sociodemographic variables, the reciprocity norm has a significant positive effect on residents’ willingness to participate at the 1% statistical level, and the standardised path coefficient is 0.278. Hypothesis H1 is supported: Reciprocity norm promote social cooperation with good expectations of mutual benefit, which is true in residents’ participation in food safety governance. Social engagement has a significant positive effect on residents’ willingness to participate at the significance level of 5%, and the standardised path coefficient is 0.108. Thus, hypothesis H2 is supported. Broad social engagement gives residents a stronger sense of belonging to the environment they live in and is more likely to create a sense that everyone is responsible for food safety governance and therefore higher participation enthusiasm in food safety governance. Institutional trust has a significant positive effect on residents’ willingness to participate at the 10% statistical level, and the standardised path coefficient is 0.093. Therefore, hypothesis H3 is supported. If residents lack trust in local governments and regulators, then they will feel the asymmetry of costs and benefits, which will inhibit their willingness to participate to a certain extent. Conversely, residents with higher levels of trust in the government perceived greater benefits of participation. Interpersonal trust has a negative effect on residents’ willingness to participate, but it is not significant. Hypothesis H4 is not supported. This result is similar to the conclusion drawn by Cai and Zhu that interpersonal trust harms farmers’ willingness to participate in the supply of water conservancy facilities [61], and the effect is not significant. The possible explanation is that residents with higher special trust are more likely to be influenced by family and friends; thus, they have a stronger sense of ‘wait and see’, which holds back their willingness to participate.

Political efficacy has a significant positive effect on residents’ willingness to participate at the 1% statistical level, and the standardised path coefficient is 0.208. Therefore, Hypothesis H5 is supported. The political efficacy reflecting residents’ democratisation consciousness positively affects residents’ willingness to participate. Reciprocity norm has a significant positive effect on political efficacy at the 10% statistical level, and the standardised path coefficient is 0.089. Therefore, hypothesis H6 is supported. Social engagement and institutional trust both have significant positive effects on political efficacy at the statistical level of 1%, and the standardised path coefficients are 0.156 and 0.494, respectively. Therefore, hypothesis H7 and H8 are supported.

Interpersonal trust has a positive effect on political efficacy, but it is not significant; therefore, hypothesis H9 is not supported. A possible reason is that the interpersonal trust in this study is only for close relatives, and people with high special trust have lower awareness of collective activities [62] and a weaker perception of political effectiveness.

Among the sociodemographic variables, age has a significant positive effect on residents’ willingness to participate at the 5% statistical level, and the standardised path coefficient is 0.107, indicating that the older the residents are, the stronger their willingness to participate. Gender has a significant positive effect on residents’ willingness to participate at the 10% statistical level, and the standardised path coefficient is 0.058. This result indicates that compared with men, women are more inclined to participate in food safety governance. Household income has a significant negative effect on residents’ willingness to participate at the 1% statistical level, and the standardised path coefficient is −0.096, indicating that residents with higher household income have lower willingness to participate. The possible explanation is that families with higher household incomes are more inclined to buy superior-quality food, and the possibility of the passive consumption of unsafe food is low. Their risk perception degree is low [63], so their willingness to participate is weak. Education level has a positive effect on residents’ willingness to participate, but it is not significant.

### 4.5. The Intermediary Effect Test of Political Efficacy

According to the estimated results in Table 5, reciprocity norm, social engagement and institutional trust have direct and significant positive effects on residents’ willingness to participate, and political efficacy plays a partial intermediary effect. The results of the direct effect, indirect effect and total effect are shown in Table 6. From the total effect, reciprocity norm has the greatest effect on residents’ willingness to participate, reaching 0.297, and the effects of social engagement and institutional trust on residents’ willingness to participate are 0.140 and 0.196, respectively. Social capital can provide the necessary political resources for residents to a certain extent, which has an impact on the sense of political efficacy and then affects the behaviour of political participation [23]. Residents with a higher level of institutional trust have more positive psychological expectations of the government’s response, form a higher sense of political efficacy and are more willing to participate in food safety governance. Residents with high levels of reciprocity norm and social engagement often have a strong sense of community [23], which can stimulate residents to express their interest demands actively and participate in food safety governance consciously.

In summary, residents’ willingness to participate in food safety governance can be explained by the joint influence of social capital and political efficacy. Social capital, as the link between residents and the social environment, restricts and modifies residents’ participation. Political efficacy is residents’ judgment of their political ability combined with previous experience. Combining social capital and political efficacy is also in line with the social principles of Chinese residents [41].

## 5. Fuzzy-Set Qualitative Comparative Analysis

After the empirical analysis above, the theoretical assumptions of social capital, political efficiency and residents’ willingness to participate were verified. However, the causes of the result are often dependent and multiple. Empirical research based on traditional statistical technology cannot reflect the multiple-conjunctural causation. Qualitative comparative analysis (QCA) with the overall research perspective is an effective method for solving the above problems [51]. Pappas and Woodside pointed out that SEM and qualitative comparative analysis are complementary, and we can use them to look at one phenomenon from different angles [13]. Therefore, we adopted fsQCA to further analyse the relationships between residents’ high willingness to participate and its antecedents, which include reciprocity norm, social engagement, institutional trust, political performance, gender, age and family income.

### 5.1. Calibration of Variables

Firstly, we calculated the averages of social engagement, reciprocity norm, institutional trust, political efficiency and residents’ willingness to participate. The average data were calibrated on the basis of the 5%, 95% and 50% of the intersection of the standards proposed by Ragin [51]. Gender, age and family income variables were directly calibrated according to the standards. After the calibration, the necessary analysis for each single antecedent condition was conducted. According to Schneider and Wagemann, the necessary condition’s consistency rate needs to reach the standard threshold of 0.9 [64]. The consistency rate of a single condition in this study did not reach 0.9; thus, seven antecedent variables were regarded as non-essential conditions. Therefore, multiple previous factors needed to be combined for configuration analysis. This study adopted fsQCA3.0 software to analyse residents’ willingness to participate in food safety governance. In the fsQCA, the consistency threshold was set to 0.85 and the acceptable case settings was set to 5. The analysis results are shown in Table 7. The overall consistency was 0.86 (the standard threshold is 0.8), and the overall coverage rate was 0.47 (the standard threshold is 0.45), indicating that fsQCA was suited to the data from this study.

### 5.2. FsQCA Results

Table 7 shows the effects of social capital and political efficacy on residents’ willingness to participate according to fsQCA. The four configurations formed by social capital, political efficacy and demographic variables are the preconditions for achieving residents’ high willingness to participate. The four solutions are as follows:

Solution 1 is ‘RN●InsT●PE●~Gender●~Income’. This solution indicates that 19% of residents who are willing to participate in food safety governance are men who have high reciprocity norm, institutional trust and political efficacy and low household incomes. Similar to previous studies, families with low incomes have fewer choices in food quality when consuming food and are more likely to encounter unsafe food [63]. Compared with women, men are more likely to participate in political activities and collective actions, become members of political parties [65] and have a higher sense of political efficacy [66].

Solution 2 is ‘RN●SE ●InsT●PE●~Gender●~Age’. This solution indicates that 15% of residents who are willing to participate in food safety governance are young men with high reciprocity norm, social engagement, institutional trust and political efficacy. Compared with the elderly, young people have a stronger perception of food safety risks [63]. Studies have confirmed that the age group under the age of 30 has the highest degree of participation in rights protection struggles [67].

Solution 3 is ‘SE●InsT●PE●Gender●~Income’. This solution indicates that 21% of residents who are willing to participate in food safety governance are women with high social engagement, institutional trust and political efficacy and lower household income. Women are more inclined to participate when the mode of participation is easy to integrate into daily life, such as informal activities. When they have the same political efficacy as men, the degree of women’s participation in political activities will increase [65].

Solution 4 is ‘RN●~SE●InsT●Gender●Age●~Income’. This solution indicates that 14% of residents who are willing to participate in food safety governance are older women with high reciprocity norm and institutional trust and lower social engagement and household income. With increasing age, women are more likely to be politically active than men [65].

In summary, the results of fsQCA confirmed that reciprocity norm, institutional trust and political efficacy were still the core elements for predicting residents’ willingness to participate. However, social engagement became less important and a non-core element; sociodemographic variables remained indispensable factors in analysing residents’ willingness to participate. fsQCA confirmed the influence of social capital and political efficacy on residents’ willingness to participate, and the four solutions are the antecedents of residents’ willingness to participate.

## 6. Conclusions and Policy Recommendations

### 6.1. Conclusions

In this study, SEM could be used to explore the effects of social capital and political efficacy on residents’ willingness to participate in food safety governance. The main conclusions are as follows.

Firstly, residents’ social capital and political efficacy affect their willingness to participate in food safety governance. Reciprocity norm, social engagement and institutional trust have positive effects. Reciprocity norm has a greater effect, which shows that the reciprocity norm plays an important role in the field of public governance of food safety governance. Moreover, reciprocity norm, social engagement and institutional trust affect residents’ willingness to participate through political efficacy.

Secondly, four solutions trigger residents’ high willingness to participate. The configurations of S1–S4 are ‘RN●InsT●PE●~Gender●~Income’, ‘RN●SE●InsT●PE●~Gender●~Age’, ‘SE●InsT●PE●Gender●~Income’ and ‘RN●~SE●InsT●Gender●Age●~Income’. The explanation strength of solution 3 is greater than that of the other solutions. The configuration with political efficacy as the core element is a favourable antecedent for promoting residents’ willingness to participate. The fsQCA and SEM analysis results are similar, and the different combinations of social capital and political efficacy are important antecedents of residents’ willingness to participate.

### 6.2. Policy Recommendations

According to the above conclusions, to promote residents’ participation in food safety management, we can start from the following aspects.

Firstly, increase residents’ willingness to participate in food safety governance by enhancing residents’ political efficacy. The government’s response to public opinions and complaints affects the public’s sense of political efficacy; hence, the government should further unblock the public’s food safety complaints and reporting channels, respond promptly to public food safety complaints and reports and enhance the public’s satisfaction and sense of gain in participating in food safety governance. Additionally, improving public political literacy; strengthening the publicity and education on laws, regulations, guidelines and policies related to food safety for residents; enhancing policy recognition; and removing obstacles to residents’ participation in food safety governance are necessary.

Secondly, residents’ social capital, especially reciprocity norm and institutional trust, must be cultivated. It is essential to improve and cultivate residents’ social capital, form social norms of mutual trust and reciprocity norm and a social atmosphere of honest cooperation, promote residents’ participation in social activities and enhance the level of institutional trust. The specific ways are as follows: society should encourage developing social organisations such as food safety management and rights protection associations and improve the effective interactions between residents and organisations, to reduce the cost of institutional arrangements; additionally, it is necessary to improve residents’ trust in the government in food safety, such as enhancing the transparency of law enforcement of local regulatory departments, and to provide timely and correct food safety information and implementing policies related to food safety.

Thirdly, the government should attach importance to the intermediary effect of political efficacy in social capital and willingness to participate and form good co-governance environments. While cultivating social capital and political efficacy, we should pay attention to the combined superposition effect between them. Moreover, we should form good social co-governance environments for food safety through various means such as education and publicity to promote residents’ willingness to participate in the governance of food safety. We can rely on existing grassroots organisations, such as communities, village committees, neighbourhood committees, and organisations that are accessible to residents in their lives; establish a mutual aid and reciprocity food safety governance participation platform; give play to the mobilisation mechanism of grassroots organisations to residents; cultivate social capital; stimulate political efficacy; and form a good cycle of residents’ participation in food safety governance.

### 6.3. Limitations

This study has limitations. Firstly, the measurement of an interpersonal trust structure is slightly different from the standard threshold of effectiveness. The hypothesis of interpersonal trust is not supported in this study; future research needs to improve the measurement method of interpersonal trust. Secondly, this study only used data from 16 cities in Shandong Province, China, due to data availability. The applicability of the findings needs to be verified with more extensive data. Thirdly, in future research, it is of theoretical and practical significance to further explore what impacts the current food safety governance policies have brought about on potential participants in terms of the costs and benefits and how social capital can change their costs and benefits, so as to address the collective action dilemma.

## Figures and Tables

**Table 1:** Sociodemographic characteristics.

		Frequency (n)	Percentage (%)
Gender	Male	324	45.4
	Female	390	54.6
Age	<20 years old	134	18.8
	20–29 years old	189	26.5
	30–39 years old	141	19.8
	40–49 years old	147	20.6
	50–59 years old	61	8.5
	≥60 years old	42	5.8
Annual Household Income	USD < 7415	74	10.4
	USD 7415–14,830	257	36.0
	USD 14,830–44,490	284	39.8
	USD 44,490–74,150	71	9.9
	USD > 74,150	28	3.9
Education	Elementary School and Below	15	2.0
	Junior High School	102	14.3
	High School or Secondary School	159	22.3
	College or Undergraduate	411	57.6
	Postgraduate	27	3.8

Note: The range of annual household income includes the lower bound but not the upper bound.

**Table 2:** The result of confirmatory factor analysis and internal consistency evaluation (*N* = 714).

Construct	Items	Loading	CR	AVE
Willingness to Participate (WP)	WP-1	0.875	0.913	0.726
	WP-2	0.907		
	WP-3	0.838		
	WP-4	0.782		
Social Engagement (SE)	SE-1	0.740	0.815	0.594
	SE-2	0.782		
	SE-3	0.790		
Institutional Trust (InsT)	IT-1	0.839	0.734	0.917
	IT-2	0.861		
	IT-3	0.871		
	IT-4	0.855		
Reciprocity Norm (RN)	RN-1	0.799	0.816	0.598
	RN-2	0.816		
	RN-3	0.699		
Interpersonal Trust (IntT)	IT-5	0.880	0.680	0.432
	IT-6	0.521		
	IT-7	0.501		
Political Efficacy (PE)	PE-1	0.620	0.813	0.523
	PE-2	0.719		
	PE-3	0.691		
	PE-4	0.845		

**Table 3:** Correlation coefficients of constructs and discriminant validity.

	SE	InsT	RN	IntT	PE	WP
SE	**0.771**					
InsT	0.310	**0.958**				
RN	0.290	0.403	**0.773**			
IntT	0.349	0.422	0.679	**0.657**		
PE	0.375	0.565	0.395	0.442	**0.723**	
WP	0.249	0.326	0.436	0.300	0.369	**0.853**

Note: The diagonal (bold) elements are the square roots of AVEs, and the off-diagonal elements are the correlations among constructs.

**Table 4:** Evaluation index system and fitting results of overall fit of the structural equation model.

Index			Evaluation Standard	Fitted Value	Result
Absolute Fit Indexes	χ^2^/df	Normed Chi-square	<5	2.754	Ideal
	GFI	Goodness of Fit Index	>0.9	0.932	Ideal
	AGFI	Adjusted Goodness of Fit Index	>0.9	0.906	Ideal
	RMR	Root Mean Square Residual	<0.05	0.191	General
Relative Fit Indexes	NFI	Normed Fit Index	>0.9	0.927	Ideal
	RFI	Relative Fit Index	>0.9	0.907	Ideal
	IFI	Incremental Fit Index	>0.9	0.952	Ideal
	TLI	Tucker-Lewis Index	>0.9	0.939	Ideal
	CFI	Comparative Fit Index	>0.9	0.952	Ideal
Information Indexes	PGFI	Parsimonious Goodness of Fit Index	>0.5	0.674	Ideal
	PNFI	Parsimonious Normed Fit Index	>0.5	0.726	Ideal

**Table 5:** Hypothesis test results.

Hypotheses	Paths	Support for Hypotheses
H1	RN -> WP	Yes
H2	SE -> WP	Yes
H3	InsT -> WP	Yes
H4	IntT -> WP	No
H5	PE -> WP	Yes
H6	RN -> PE	Yes
H7	SE -> PE	Yes
H8	InsT -> PE	Yes
H9	IntT -> PE	No
--	Age ->WP	significant
--	Gender -> WP	significant
--	Household Income -> WP	significant
--	Education -> WP	insignificant

**Table 6:** Standardised results of direct, indirect and total effects.

Paths Relationship	Direct Effect	Indirect Effect	Total Effect
RN -> PE -> WP	0.278	0.019	0.297
SE -> PE -> WP	0.108	0.032	0.140
InsT -> PE -> WP	0.093	0.103	0.196

**Table 7:** FsQCA findings.

Configuration	Solutions			
	S1	S2	S3	S4
RN	⬤	⬤	--	⬤
SE	--	●	●	⊗
InsT	⬤	⬤	●	⬤
PE	⬤	⬤	⬤	--
Gender	⊗	⊗	●	●
Age	--	⊗	--	●
Income	⊗	--	⊗	⊗
Consistency	0.880	0.920	0.860	0.860
Raw Coverage	0.190	0.150	0.210	0.140
Unique Coverage	0.070	0.030	0.110	0.040
Overall Solution Consistency	0.860			
Overall Solution Coverage	0.470			

Note: Table 7 shows the intermediate solutions calculated by the fsQCA software, where black circles (⬤) indicate the presence of a condition, and circles with ‘x’ (⊗) indicate its absence. Large circle; core condition, Small circle; peripheral condition, -- indicates that is ‘don’t care’ condition.

## Data Availability

The data presented in this study are available on request from the corresponding author. The data are not publicly available due to privacy restrictions.

## Appendix A

**Table A1:** Constructs and Measurement Items.

Construct	Items	Source
Willingness to Participate (WP)	(1) I am willing to participate in food safety supervision.	[43,44]
	(2) I will consider participating in the social co-governance of food safety.	
	(3) I am willing to encourage others to participate in the governance of food safety.	
	(4) I am willing to take practical actions to support food safety co-governance.	
Social Engagement (SE)	(5) I often participate in the activities of informal groups (villagers’ associations, interest associations, etc.).	[45,46]
	(6) I often participate in community volunteer activities.	
	(7) I often participate in various activities carried out by the community (health lectures, cultural and sports activities, etc.).	
Institutional Trust (InsT)	(8) Regulators can provide correct food safety information.	[47]
	(9) The regulatory authorities can handle food safety consultations, complaints, and reports on time.	
	(10) Local governments can implement food-safety-related policies based on public opinion.	
	(11) Local regulatory authorities can enforce the law openly and impartially.	
Reciprocity Norm (RN)	(12) When relatives are in trouble, I will take the initiative to help.	[45,48]
	(13) When neighbours and friends are in trouble, I will take the initiative to help.	
	(14) When strangers are in trouble, I will take the initiative to help.	
Interpersonal Trust (IntT)	(15) I trust my friends.	[46]
	(16) I trust my family.	
	(17) I trust people who live in the same neighbourhood.	
Political Efficacy (PE)	(18) I can participate in political activities.	[49]
	(19) Suggestions to the government will be adopted.	
	(20) Government officials will attach importance to citizens’ attitudes and views.	
	(21) There are ways to let government leaders know your suggestions to the government.	
